# Chemoresistance in the era of immunotherapy: challenges and novel combinatorial strategies for digestive system tumors

**DOI:** 10.3389/fimmu.2026.1800910

**Published:** 2026-04-22

**Authors:** Lu Yang, Di Zhou, Ke Cao, Dan Mao, Chenxi Wang, Tao Wei, Yu Deng, Zihan Yi, Wenhui Li, Zhengting Chen

**Affiliations:** 1 Department of Radiation Oncology, The Third Affiliated Hospital of Kunming Medical University (Yunnan Cancer Hospital, Peking University Cancer Hospital Yunnan), Kunming, China; 2 Kunming Medical University, Kunming, China; 3 Key Laboratory of Cancer Research, The Third Affiliated Hospital of Kunming Medical University (Yunnan Cancer Hospital, Peking University Cancer Hospital Yunnan), Kunming, China; 4 Phase I Clinical Trial Ward, The Third Affiliated Hospital of Kunming Medical University (Yunnan Cancer Hospital, Peking University Cancer Hospital Yunnan), Kunming, China

**Keywords:** chemoresistance, combination therapy, digestive system tumors, immune checkpoint inhibitors, immunogenic cell death, tumor microenvironment

## Abstract

The prognosis for late-stage digestive system tumors is poor, largely due to the development of chemotherapy resistance. Although immunotherapy, particularly immune checkpoint inhibitors, has transformed the treatment landscape for some patients, strategies to further enhance the efficacy of combination therapies are still lacking, and the underlying mechanisms remain incompletely understood. To systematically address these therapeutic challenges and explore potential solutions, this review delineates the key mechanisms driving chemoresistance in digestive system tumors. It encompasses both cell-intrinsic mechanisms—such as enhanced drug efflux and DNA repair pathways—and extrinsic factors mediated by the tumor microenvironment (TME), including immune cell infiltration and metabolic reprogramming. A special emphasis is placed on the dual immunomodulatory roles of chemotherapy-induced immunogenic cell death (ICD) and its remodeling impact on the immune landscape. Given the considerable heterogeneity across digestive system cancers—including gastric, colorectal, and hepatic malignancies—the review also synthesizes recent advances in innovative combination strategies. These include immunochemotherapy, oncolytic virus, targeting of cancer stem cells (CSCs), epigenetic modulation, and nanoparticle-based drug delivery systems. Ultimately, this work aims to offer a theoretical foundation and strategic directions to overcome clinical drug resistance and advance precision oncology in digestive system tumors.

## 1 Introduction

Digestive system neoplasms, including esophageal, gastric, colorectal, pancreatic, and hepatocellular carcinomas, remain a leading cause of cancer-related mortality worldwide (1). Despite advances in screening and surgical techniques, their rising incidence and poor prognosis continue to pose a major public health challenge (2). Chemotherapy remains the backbone of treatment for patients with advanced disease. However, the persistent emergence of multidrug resistance (MDR) often leads to therapeutic failure and disease recurrence, representing a major obstacle in clinical management. In recent years, immunotherapy, particularly immune checkpoint inhibitors (ICIs) targeting the PD-1/PD-L1 axis, has reshaped the treatment landscape for a subset of digestive system tumors and provided durable clinical benefit (3). Nevertheless, the overall response rate remains limited because both primary and acquired resistance frequently compromise its efficacy (4).

Emerging evidence suggests that chemoresistance and immune evasion are not isolated processes, but are closely intertwined within the tumor microenvironment (TME). The TME is a highly heterogeneous and dynamic ecosystem composed of immune cells, cancer-associated fibroblasts (CAFs), and extracellular matrix components. Together, these elements create a sanctuary that supports tumor survival (5). Within this niche, tumor-associated macrophages (TAMs), predominantly of the M2 phenotype, establish an immunosuppressive barrier by secreting inhibitory cytokines. They also promote therapeutic resistance through angiogenesis and matrix remodeling (6). At the same time, cancer stem cells (CSCs) are increasingly recognized as a major source of tumor recurrence and immune evasion. These cells are characterized by self-renewal, pluripotency, and intrinsic resistance to cytotoxic agents (7, 8). In addition, metabolic reprogramming, such as enhanced fatty acid oxidation and aberrant branched-chain amino acid metabolism, increases tumor plasticity (9, 10). Epigenetic dysregulation, including DNA methylation, histone modification, and non-coding RNA modulation, further strengthens this adaptive capacity (11, 12). Together, these alterations allow tumor cells to dynamically adapt to therapeutic pressure while simultaneously orchestrating immune escape.

This convergence of resistance mechanisms highlights a profound crosstalk between chemotherapy and the immune system. Conventional chemotherapy exerts a double-edged effect. On the one hand, it can stimulate antitumor immunity by inducing immunogenic cell death (ICD) and enhancing antigen presentation. On the other hand, it may paradoxically trigger compensatory immunosuppressive feedback. This process may select for aggressive, low-immunogenic subclones and induce the upregulation of checkpoint molecules such as PD-L1 (13). Similarly, immune editing can impose selective pressure that drives tumor evolution toward a chemoresistant phenotype. Together, these bidirectional interactions provide a strong theoretical rationale for shifting from monotherapy to rationally designed combination strategies.

Current translational research increasingly focuses on combined intervention strategies aimed at dismantling the multilayered barriers of MDR and the immunosuppressive TME. A broad range of innovative approaches is under preclinical and clinical investigation, including synergistic chemo-immunotherapy, intelligent nanoparticle-based delivery systems, CSC-targeting agents, epigenetic reprogrammers, and metabolic modulators. Among these, two strategies have shown particular promise in restoring therapeutic sensitivity and reversing resistance: nanotechnology-based spatiotemporal co-delivery of chemotherapeutics and immunomodulators, and epigenetic drugs that reprogram cold tumors into immunologically hot microenvironments.

This review provides a comprehensive overview of the current understanding of the crosstalk between chemoresistance and immune evasion in digestive system tumors. In addition to summarizing the existing literature, we critically discuss the controversies and challenges associated with current therapeutic paradigms. We also highlight recent advances in emerging combinatorial strategies, including CSC targeting, metabolic reprogramming, oncolytic virotherapy, epigenetic regulation, and nanomedicine. Ultimately, we aim to provide a conceptual basis for overcoming clinical resistance and advancing precision oncology.

## 2 Mechanisms of chemoresistance: the dual challenge of intrinsic adaptation and microenvironmental shielding

### 2.1 Cell-intrinsic mechanisms: converging on immune evasion

**Figure 1:**
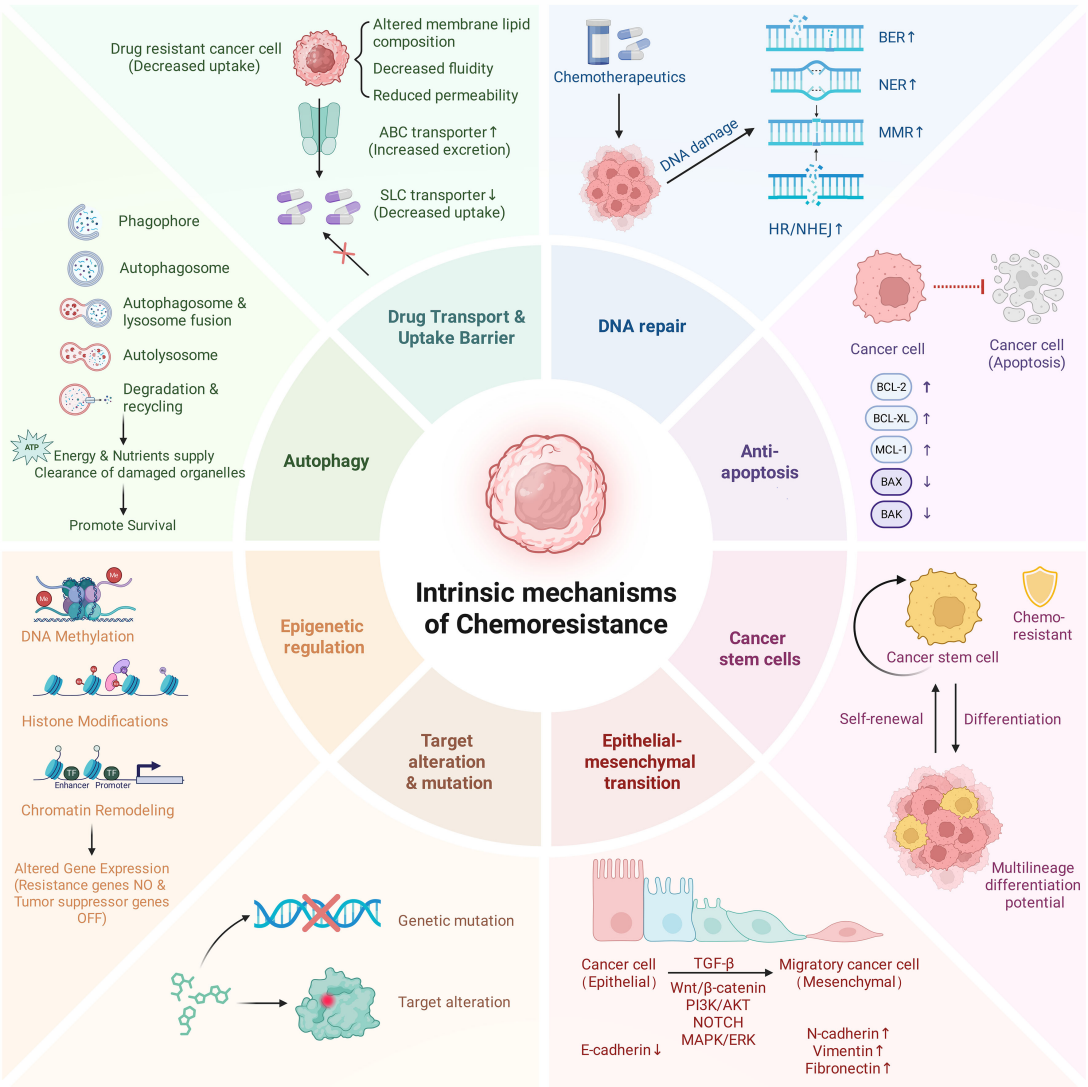
Intrinsic chemoresistance mechanisms drive systemic immune tolerance. Eight molecular adaptations (outer panels) function synergistically to shield the cancer cell (center) from immune surveillance, resulting in three immunosuppressive outcomes: (1) Immunologically Quiescent Phenotype: Mechanisms including hyper-active DNA repair, structural target alterations, and epigenetic reprogramming converge to minimize neoantigen load and silence immunogenicity. (2) Plasticity-Driven Immune Exclusion: The acquisition of stem-like properties (CSCs) and mesenchymal phenotypes (EMT) mediates the downregulation of MHC-I and the upregulation of immune checkpoints (e.g., PD-L1). (3) Failure of Immunogenic Cell Death (ICD): Resistance to apoptosis, cytoprotective autophagy, and transporter-mediated efflux blockade the release of danger signals (DAMPs) and induce T-cell toxicity. Collectively, these intrinsic barriers dictate the “immune-cold” nature of chemoresistant digestive system tumors. Figure created with BioRender.com.

The acquisition of chemotherapy resistance in digestive system neoplasms is fundamentally driven by a series of adaptive evolutionary changes. However, emerging evidence suggests that these alterations function not merely as a survival shield against cytotoxic stress, but as a coordinated program that fundamentally reshapes the tumor’s immunological identity. As illustrated in **Figure 1**, the diverse intrinsic mechanisms of resistance converge into three distinct immunological phenotypes that actively thwart surveillance.

First, mechanisms maintaining genomic integrity and epigenetic stability—such as hyper-active DNA repair and transcriptional silencing—prevent the accumulation of neoantigens, rendering the tumor immunologically quiescent (“Silent”). Second, cellular plasticity driven by stemness (CSCs) and epithelial-mesenchymal transition (EMT) dynamically remodels surface markers (e.g. MHC-I loss, PD-L1 upregulation), leading to adaptive immune exclusion (“Invisible”). Third, the subversion of cell death pathways—through apoptosis resistance, autophagy, and drug efflux—dismantles the signals required for Immunogenic Cell Death (ICD), actively suppressing the priming of innate and adaptive immunity. Thus, intrinsic chemoresistance is inextricably linked to the establishment of systemic immune tolerance.

#### 2.1.1 Genomic stability and antigen depletion: the immunologically quiescent phenotype

Chemoresistance is frequently underpinned by mechanisms that strictly maintain genomic integrity, a survival advantage that inadvertently renders the tumor immunologically quiescent. While classical chemotherapy aims to induce catastrophic DNA damage, resistant clones often exhibit a “hyper-repair” phenotype. By rapidly resolving DNA lesions via upregulated Nucleotide Excision Repair (NER) or Homologous Recombination (HR) pathways (14), these cells prevent the accumulation of somatic mutations. This proficient repair directly restricts the tumor mutational burden (TMB) and the subsequent generation of neoantigens, creating a paucity of T-cell targets that characterizes the “immune-cold” phenotype (15, 16). Conversely, defects in these pathways (e.g., dMMR/MSI-H) lead to genomic instability and abundant neoantigen release, triggering cGAS-STING activation (17, 18); however, resistant tumors frequently restore these pathways (e.g., via BRCA reversion mutations) to regain both genomic stability and immune invisibility (19, 20).

This loss of immunogenicity is further reinforced by epigenetic silencing. Beyond preserving the DNA sequence, resistant tumors utilize epigenetic reprogramming—mediated by DNA methyltransferases (DNMTs) and histone deacetylases (HDACs)—to transcriptionally suppress immunogenic elements (21, 22). A critical mechanism involves the silencing of endogenous retroviruses (ERVs) and Cancer-Testis Antigens (CTAs). Under basal conditions, the transcription of these repetitive elements mimics viral infection, activating the cGAS-STING pathway and Type I interferon signaling (the “viral mimicry” effect) (23, 24). However, in chemoresistant phenotypes, hypermethylation or histone deacetylation suppresses ERV expression, thereby preventing intrinsic immune sensing and maintaining a non-inflamed microenvironment.

Concurrently, specific genomic driver mutations actively sculpt this immunosuppressive landscape. Dysregulation of key oncogenes and tumor suppressors does not merely drive proliferation but actively dampens immune recognition. Mutant p53, a hallmark of resistance, actively suppresses the cGAS-STING pathway, blocking the sensing of cytosolic DNA and inhibiting Type I interferon production (25). Similarly, mutant KRAS orchestrates the establishment of an exclusionary microenvironment by driving the secretion of GM-CSF to recruit Myeloid-Derived Suppressor Cells (MDSCs) (26, 27). Furthermore, the oncogene c-Myc directly upregulates the “don’t eat me” signal CD47 and the checkpoint PD-L1, creating a double blockade against macrophage phagocytosis and T-cell cytotoxicity (28). Thus, the machinery driving intrinsic chemoresistance—comprising hyper-efficient DNA repair, epigenetic silencing, and oncogenic signaling—converges to strip the tumor of its immunogenicity, establishing a formidable barrier to immune surveillance.

#### 2.1.2 Plasticity-driven immune exclusion: CSCs and EMT

Cellular plasticity—encompassing the acquisition of stem-like properties (CSCs) and the execution of Epithelial-Mesenchymal Transition (EMT)—functions as a potent driver of both therapeutic recalcitrance and intrinsic immune evasion. Beyond their metabolic adaptability and quiescence, these plastic states are fundamentally characterized by their ability to remodel the immune synapse, effectively establishing an immune-privileged reservoir that fuels relapse.

First, plasticity is intrinsically linked to defects in antigen presentation. Cancer Stem Cells (CSCs) actively evade CD8^+^ T-cell surveillance by downregulating MHC Class I molecules and the Antigen-Processing Machinery (APM), a feature essential for their maintenance in a dormant state (29). This loss of immunogenicity is amplified during EMT. The transition to a mesenchymal phenotype is frequently accompanied by the epigenetic silencing of MHC Class I genes. Consequently, even if chemotherapy induces high loads of neoantigens, these plastic cells fail to present them on the cell surface, rendering them refractory to cytotoxic T lymphocyte (CTL) recognition (30).

Second, plastic cells actively upregulate inhibitory checkpoints to paralyze innate and adaptive immunity. In CSCs, the overexpression of CD47 serves as a critical “don’t eat me” signal, preventing phagocytosis by macrophages and dendritic cells (31). Concurrently, the transcriptional network driving EMT directly rewires adaptive immune resistance. It is now established that key EMT-inducing transcription factors, specifically ZEB1 and Snail, act as direct transcriptional activators of the CD274 (PD-L1) promoter. This molecular bridge ensures that as cells acquire motile and chemoresistant traits, they simultaneously acquire the capacity to exhaust infiltrating T cells (32, 33).

Third, this plasticity orchestrates the formation of an exclusionary microenvironment. Both CSCs and mesenchymal-like tumor cells exhibit a pro-inflammatory but immunosuppressive secretory profile, characterized by high levels of TGF-β and IL-10. These factors actively recruit regulatory T cells (Tregs) and M2-polarized macrophages while driving stromal fibrosis (34–36). This combination of immunosuppressive cell accumulation and matrix stiffening creates a physical and chemical barrier that excludes effector lymphocytes from the tumor core, solidifying the “immune-excluded” phenotype (37, 38). Thus, cellular plasticity functions as a central mechanism where intrinsic chemoresistance converges with systemic immune tolerance.

#### 2.1.3 Defective death signaling and failure of immunogenic cell death

The failure of chemotherapy to trigger a robust immune response is frequently attributable to the active subversion of cell death pathways. For chemotherapy to be effective in the long term, it must induce Immunogenic Cell Death (ICD)—a specific mode of regulated cell death that releases Damage-Associated Molecular Patterns (DAMPs) to prime the immune system. However, resistance mechanisms systematically dismantle this process, rendering the tumor immunologically quiescent.

First, apoptosis resistance prevents the release of danger signals. The Bcl-2 family serves as the central rheostat, where overexpression of anti-apoptotic members (e.g., Bcl-2, Bcl-xL, and MCL-1) preserves mitochondrial outer membrane permeabilization (MOMP) (39). Crucially, this preservation prevents the cytosolic leakage of mitochondrial DNA (mtDNA), thereby silencing the cGAS-STING viral-sensing pathway (40, 41). Without this signal, the downstream production of Type I interferons is abrogated, rendering the cell death process tolerogenic rather than immunogenic. Furthermore, upstream signaling nodes like PI3K/Akt and JAK2/STAT3 often concurrently upregulate PD-L1, creating a dual mechanism that blocks apoptosis while simultaneously exhausting T cells (42, 43).

Second, cytoprotective autophagy degrades immune markers. Under therapeutic stress, autophagy is hijacked not only to recycle nutrients and raise the apoptotic threshold (44, 45), but also to actively dismantle the antigen presentation machinery. Recent evidence suggests that hyperactive autophagy selectively degrades MHC Class I molecules via the autolysosomal pathway, further impairing antigen presentation and blinding CD8^+^ T cells to the presence of the tumor (46).

Third, transporter-mediated drug efflux creates a hostile niche. While ABC transporters (e.g., P-gp/ABCB1) canonically reduce intracellular drug concentrations to ensure survival (47), their upregulation exerts a detrimental “bystander effect” on immunity. Chemotherapeutics extruded from tumor cells accumulate in the interstitial fluid of the Tumor Microenvironment (TME). Since tumor-infiltrating lymphocytes (TILs) lack the robust efflux machinery possessed by resistant tumor cells, this high extracellular drug concentration can induce T-cell apoptosis or functional exhaustion, reinforcing an immunosuppressive state (48, 49). Additionally, specific transporters like ABCC4 facilitate the efflux of immunosuppressive signaling molecules (e.g., prostaglandins), further dampening the immune response (50, 51). Thus, these survival mechanisms cooperate to ensure that even when tumor cells are stressed, they fail to elicit—and actively suppress—an effective immune response.

### 2.2 Extrinsic remodeling: the TME as an immunological fortress

**Figure 2:**
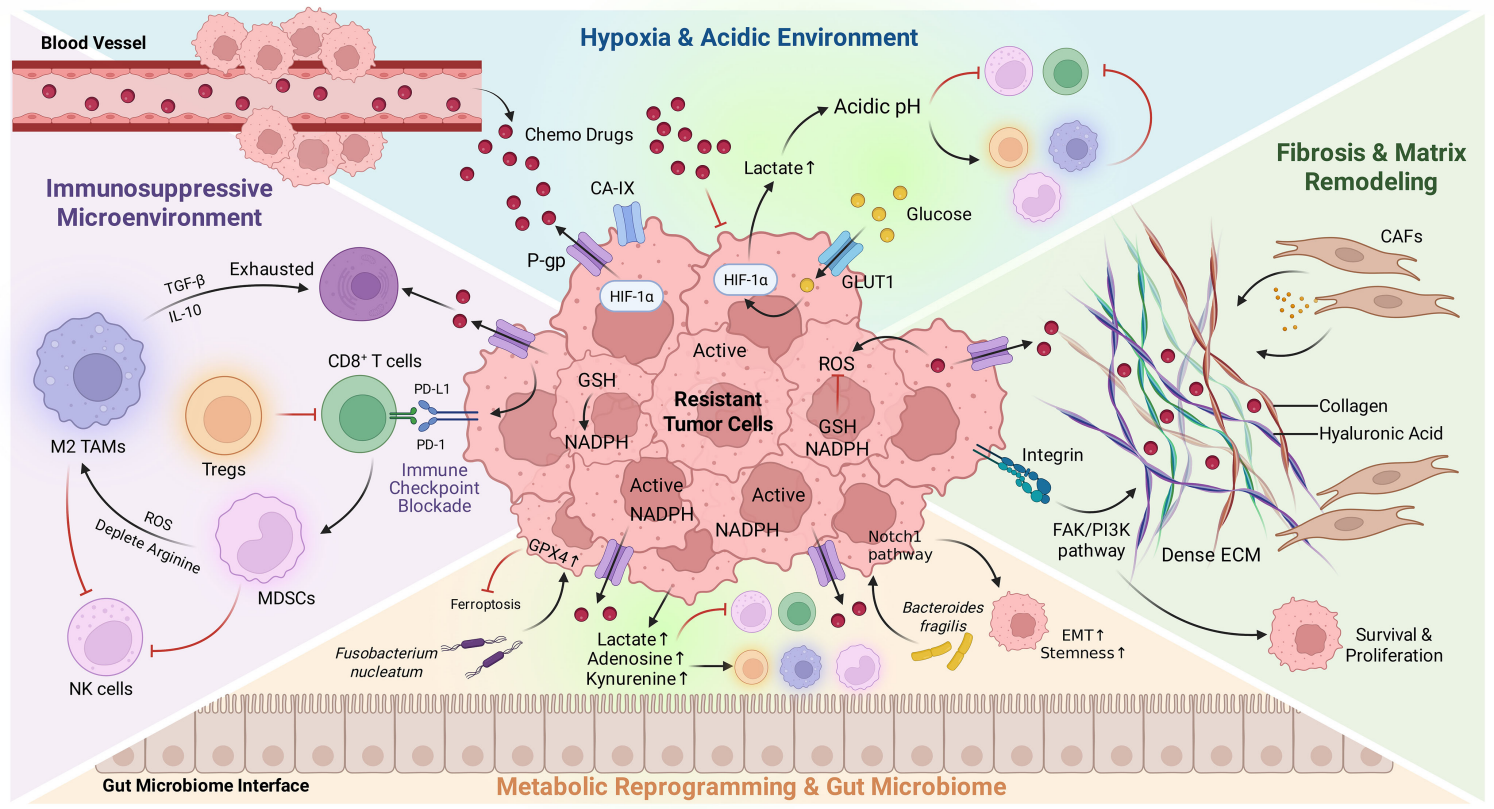
Extrinsic remodeling of the Tumor Microenvironment (TME) into an immunological fortress. The schematic illustrates how resistant tumors construct an extrinsic sanctuary against therapy through three functional barriers: (1) The Metabolic Barrier (upper panels): Driven by hypoxia and acidosis, HIF-1α signaling restricts drug influx via P-gp and accumulates immunosuppressive metabolites (lactate, adenosine, kynurenine) that impair T-cell cytotoxicity. (2) The Cellular Barrier (left panels): Chemotherapy-induced damage recruits regulatory cells (M2-TAMs, Tregs, MDSCs) via CCL2. These populations confer survival signals and enforce tolerance through the PD-1/PD-L1 axis. (3) The Physical (right) and Biotic Barrier (bottom): Fibrosis creates a dense matrix promoting “immune exclusion” and integrin-FAK signaling. Concurrently, the gut microbiome (e.g., F. nucleatum) inhibits ferroptosis and suppresses immunity via TIGIT binding. Collectively, these extrinsic forces converge to shield the tumor from both cytotoxic drugs and immune surveillance. Figure created with BioRender.com.

The Tumor Microenvironment (TME) functions not merely as a passive structural scaffold but as an active co-conspirator in therapeutic recalcitrance. While intrinsic mechanisms allow individual cells to survive, the TME constructs an extrinsic sanctuary that shields malignant clones from both cytotoxic insults and immune surveillance. As illustrated in **Figure 2**, chemotherapy often triggers a “wound-healing” response that is hijacked by the tumor to fortify this sanctuary. This extrinsic resistance is governed by three distinct but interconnected barriers: (1) The Metabolic Barrier: A hostile physicochemical landscape defined by hypoxia and acidosis, where the accumulation of oncometabolites (e.g., lactate, adenosine) mechanically paralyzes T-cell function while chemically impeding drug distribution. (2) The Cellular Barrier: A dynamic suppressive network where chemotherapy-induced damage signals (e.g., CCL2) actively recruit “bodyguard” populations—including MDSCs, M2-TAMs, and Tregs—to inhibit cytotoxic immunity. (3) The Physical and Biotic Barrier: A structural fortress built by dense fibrotic stroma that physically excludes effector T cells (“immune exclusion”), reinforced by a dysbiotic microbiome (e.g., F. nucleatum) that functionally disarms immune cells via checkpoint engagement. Collectively, these extrinsic forces forge a multidimensional fortress, ensuring that the tumor remains resilient to the dual attack of chemotherapy and immunotherapy.

#### 2.2.1 The metabolic barrier: hypoxia and adenosine

The physicochemical landscape of chemoresistant tumors is defined by profound hypoxia, a consequence of aberrant vasculature and rapid proliferation. This oxygen-deprived niche triggers adaptive signaling centered on Hypoxia-inducible factor-1α (HIF-1α). Crucially, HIF-1α orchestrates a dual resistance program: it sustains bioenergetics by upregulating glycolytic enzymes while simultaneously functioning as a master regulator of immune suppression, transforming the TME into a hostile metabolic desert.

A primary consequence of this hypoxic adaptation is the intensification of the Warburg effect. To survive oxygen deprivation, resistant cells shift their metabolism toward aerobic glycolysis, resulting in the excessive accumulation of lactate and the subsequent acidification of the extracellular matrix via Carbonic Anhydrase IX (CA-IX) (52). This acidic microenvironment exerts a synergistic negative impact: biologically, it paralyzes the cytolytic function of effector T cells and NK cells (53, 54) while fostering the recruitment of suppressive subsets like Tregs and M2-TAMs (55–57); pharmacologically, it creates an “ion trapping” effect that impairs the membrane permeability of weakly basic chemotherapeutics (e.g., doxorubicin), reducing their intracellular accumulation (58).

Beyond acidosis, hypoxia drives a potent “purinergic switch” to mechanically shut down anti-tumor immunity. Under hypoxic stress, HIF-1α directly upregulates the expression of the ectonucleotidases CD39 and CD73. These enzymes hydrolyze extracellular ATP—released by dying cells—into adenosine, a highly immunosuppressive metabolite. Extracellular adenosine binds to A2A receptors on infiltrating T cells, elevating intracellular cAMP levels and effectively “locking” them in an exhausted state, unable to secrete cytokines or execute cytotoxicity (59).

Complementing these active suppressive mechanisms, chemoresistant tumors enforce a “metabolic checkpoint” through nutrient depletion. Overexpression of enzymes like Indoleamine 2, 3-dioxygenase (IDO) depletes essential tryptophan to arrest T-cell proliferation while generating kynurenine to induce Treg differentiation (60, 61). Furthermore, upregulated antioxidant systems (e.g., GSH/NADPH) neutralize chemotherapy-induced ROS, inhibiting ferroptosis while protecting the immunosuppressive function of MDSCs (62–66). Thus, the metabolic landscape of resistance functions as a multi-tiered physiological barrier that simultaneously repels cytotoxic drugs and disarms the immune system.

#### 2.2.2 The cellular barrier: recruitment of suppressor cells

The establishment of an immunosuppressive cellular barrier is not merely a static feature of the tumor but a dynamic reaction frequently exacerbated by therapeutic intervention. Paradoxically, the “wound healing” response triggered by chemotherapy-induced tissue damage is hijacked by resistant tumors to recruit a phalanx of suppressive cell populations—primarily Myeloid-Derived Suppressor Cells (MDSCs), Tumor-Associated Macrophages (TAMs), and Regulatory T cells (Tregs).

The recruitment phase is orchestrated by distinct chemokine signals. In response to cytotoxic stress (e.g., paclitaxel or 5-FU), resistant tumor cells upregulate the secretion of CCL2 (MCP-1) and CSF-1, which act as potent chemoattractants for myeloid progenitors (67, 68). Upon infiltration, these cells are rapidly educated toward a suppressive phenotype (e.g., M2-TAMs) within the TME (69). This creates a “pro-tumorigenic feedback loop”: chemotherapy kills sensitive clones but simultaneously enriches the stroma with protective immune cells.

Once established, these suppressive cells erect a dual barrier against therapy. First, they provide direct survival signals to shield cancer cells. In colorectal cancer, 5-FU exposure stimulates TAMs to secrete putrescine, which inhibits the JNK/caspase-3 pathway in tumor cells, effectively blocking apoptosis (70). Similarly, M2-TAMs in resistant gastric cancer release CXCL5 to activate the PI3K/AKT/mTOR axis in tumor cells, further reinforcing intrinsic resistance (71). Second, they actively paralyze cytotoxic immunity. MDSCs suppress T-cell proliferation by depleting essential amino acids (e.g., arginine via Arginase-1) and generating nitric oxide (NO) and ROS, creating a local environment where CTLs cannot survive or function (72). Concurrently, Tregs recruited via kynurenine metabolites secrete high levels of TGF-β and IL-10, which not only dampen NK cell cytotoxicity but also drive further epithelial-mesenchymal transition (EMT) in the tumor, closing the loop of resistance (73–75).

Furthermore, this cellular network is intrinsically linked to checkpoint evasion. The accumulation of these suppressive cells correlates with the upregulation of PD-L1. In pancreatic cancer, PD-L1 expression is positively correlated with FOXP3+ Treg infiltration (76). Crucially, the PD-1/PD-L1 axis has been shown to directly enhance cisplatin resistance in GC by upregulating P-gp via PI3K/AKT signaling, establishing a direct molecular bridge between immune tolerance and multidrug resistance (77). Thus, the recruitment of suppressor cells serves as an extrinsic “bodyguard” that ensures therapeutic failure.

#### 2.2.3 The physical and microbial barrier

Beyond cellular and metabolic hurdles, the TME erects formidable physical and biotic barriers—comprising the extracellular matrix (ECM) and the gut microbiome—that physically obstruct drug delivery and functionally disarm immune effectors.

The primary physical barrier is orchestrated by Cancer-Associated Fibroblasts (CAFs). In desmoplastic tumors like PDAC and HCC, CAFs drive the excessive deposition of collagen and hyaluronan, creating a dense, stiff ECM (78). This fibrosis exerts a dual exclusionary effect. Mechanically, it elevates interstitial fluid pressure (IFP) to collapse tumor vasculature, hindering the perfusion of chemotherapeutics (79, 80). Immunologically, this stiffened matrix functions as a “physical wall” that dictates the “immune-excluded” phenotype. CAFs secrete high levels of TGF-β and crosslink collagen via enzymes like LOXL2, which not only activates the FAK/PI3K signaling axis to drive adhesion-dependent drug resistance (CAM-DR) (81, 82) but also restricts T-cell motility. Consequently, cytotoxic CD8^+^ T cells are trapped in the stroma, physically prevented from making contact with cancer cells, establishing an “immune desert” within the tumor core (83).

Complementing this physical fortress is the biotic barrier of the gut microbiome. Dysbiosis functions as a critical extrinsic modulator of resistance. Specifically, the oncogenic bacterium Fusobacterium nucleatum (F. nucleatum) acts as a sophisticated accomplice in resistance. On one hand, F. nucleatum promotes intrinsic chemoresistance by upregulating autophagy or inhibiting ferroptosis via the E-cadherin/β-catenin axis (84). On the other hand, it directly suppresses anti-tumor immunity. F. nucleatum expresses the Fap2 protein, which directly binds to the inhibitory receptor TIGIT on NK cells and T cells. This binding triggers an inhibitory signal that paralyzes the cytotoxic activity of these lymphocytes, effectively protecting the tumor from immune-mediated killing even in the presence of chemotherapy (85, 86). Furthermore, other bacteria (e.g., Gammaproteobacteria) can metabolically inactivate drugs like gemcitabine (87), further solidifying the microbiome as a critical axis of therapeutic failure.

## 3 Immunotherapy in digestive cancers: clinical heterogeneity and biological barriers

### 3.1 Current clinical landscape: heterogeneity dictated by immune phenotypes

Immune Checkpoint Inhibitors (ICIs) have revolutionized oncology by re-activating anti-tumor T cell immunity (88). However, the clinical landscape of digestive system cancers reveals a profound dichotomy: while some malignancies achieve durable remission, others exhibit intrinsic refractoriness. This variability is not random but is strictly dictated by the immunological contexture of the Tumor Microenvironment (TME). Clinically, digestive malignancies can be categorized into three distinct phenotypes — “Hot”, “Excluded”, and “Cold”— each requiring tailored therapeutic strategies. Key pivotal clinical trials defining this landscape are summarized in **Table 1**.

**Table 1:** Summary of pivotal clinical trials evaluating immune checkpoint inhibitors (ICIs) and combinatorial strategies in digestive system cancers.

Trial name	Cancer type	Regimen	Median follow-up	Primary endpoint results	Notes	References
KEYNOTE-590	EC/GEJC	Pembrolizumab + Chemo vs Chemo	58.8 months	OS: 12.3 vs 9.8 months (HR 0.72), PFS: 6.3 vs 5.8 months (HR 0.64) OS (CPS≥1): 12.7 vs 9.9 months (HR 0.70), PFS (CPS≥1): 6.3 vs 5.7 months (HR 0.61)	Pembrolizumab combined with chemotherapy shows significant survival benefit in advanced esophageal cancer.	(92, 186)
CheckMate-648	ESCC	Nivo + Chemo vs Chemo	≥13 months	OS: 13.2 vs 10.7 months (HR 0.74), PFS: 5.8 vs 5.6 months (HR 0.81) OS (PD-L1≥1%): 15.4 vs 9.1 months (HR 0.54), PFS(PD-L1≥1%): 6.9 vs 4.4 months (HR 0.65)	Nivolumab combined with chemotherapy significantly improves survival, especially in PD-L1 high expression patients.	(93)
ESCORT-1st	ESCC	Camrelizumab + TP vs Placebo + TP	≥24 months	OS: 15.6 vs 12.6 months (HR 0.70), PFS: 6.9 vs 5.6 months (HR 0.56)	Camrelizumab combined with chemotherapy shows significant survival benefit in advanced ESCC, with good safety profile.	(94, 187)
JUPITER-06	ESCC	Toripalimab + TP vs Placebo + TP	14.2 months	OS: 17.7 vs 12.9 months (HR 0.72)	Toripalimab combined with chemotherapy significantly prolongs OS, with good efficacy and tolerability.	(95)
ORIENT-15	ESCC	Sintilimab + Chemo vs Placebo + Chemo	32.2 months	OS: 17.4 vs 12.8 months (HR 0.66), PFS: 7.2 vs 5.7 months (HR 0.56) OS (CPS ≥ 10): 18.4 vs 14.5 months (HR 0.64), PFS (CPS ≥ 10): 8.3 vs 6.4 months (HR 0.58)	Sintilimab combined with chemotherapy significantly improves survival, especially in PD-L1 high expression patients.	(96, 188)
CheckMate-649	HER2-negative GC/GEJC	Nivolumab + Chemo vs Chemo	71.3 months	OS: 13.8 vs 11.4 months (HR 0.79), PFS: 7.8 vs 6.9 months (HR 0.79); OS (CPS ≥ 10): 15.0 vs 10.9 months (HR 0.68), PFS (CPS ≥ 10): 8.4 vs 5.8 months (HR 0.68)	Immunotherapy combined with chemotherapy significantly extends survival, especially in PD-L1 CPS ≥5 patients.	(97)
KEYNOTE-859	HER2-negative GC/GEJC	Pembrolizumab + Chemo vs Chemo	24.7 months	OS: 15.9 vs 12.2 months (HR 0.68), PFS: 8.1 vs 5.7 months (HR 0.65)	Pembrolizumab combined with chemotherapy significantly improves survival, particularly in the Chinese subgroup.	(98, 189)
KEYNOTE-811	HER2-positive GC/GEJC	Pembrolizumab + Trastuzumab + Chemo vs Trastuzumab + Chemo	38.4 months	OS: 20.0 vs 16.8 months (HR 0.84), PFS: 10.0 vs 8.1 months (HR 0.73)	Immunotherapy combined with trastuzumab and chemotherapy significantly improves PFS and OS.	(99)
ORIENT-16	GC/GEJC	Sintilimab + Chemo vs Chemo	18.8 months	OS: 15.2 vs 12.3 months (HR 0.77), PFS: 7.1 vs 5.7 months (HR 0.64); OS (CPS≥5): 18.4 vs 12.9 months (HR 0.66), PFS: 7.7 vs 5.8 months (HR 0.63)	Sintilimab combined with chemotherapy significantly improves survival in advanced gastric cancer, especially in PD-L1 CPS ≥5 patients.	(190)
RATIONALE-305	GC/GEJC	Tislelizumab + Chemo vs Chemo	≥36.6 months	OS: 15.0 vs 12.9 months (HR 0.79), PFS: 6.9 vs 6.2 months (HR 0.79); OS (PD-L1 ≥ 5%): 16.4 vs 12.8 months (HR 0.71), PFS (PD-L1 ≥ 5%): 7.2 vs 5.9 months (HR 0.69)	Tislelizumab combined with chemotherapy extends survival, particularly in PD-L1 ≥5% patients.	(191)
GEMSTONE-303	GC	Sugrilimab + Chemo vs Chemo	25.1 months	OS (CPS ≥ 5): 15.6 vs 12.6 months (HR 0.75), PFS (CPS ≥ 5): 7.6 vs 6.1 months (HR 0.66); OS (CPS ≥ 10): 17.8 vs 12.5 months (HR 0.64), PFS (CPS ≥ 10): 7.8 vs 5.5 months (HR 0.58)	Sugrilimab combined with chemotherapy shows significantly better survival benefits for PD-L1 CPS ≥5 patients.	(192)
KEYNOTE-177	MSI-H/dMMR mCRC	Pembrolizumab (monotherapy) vs Chemotherapy	73.3 months	OS: 77.5 vs 36.7 months (HR 0.73), PFS: 16.5 vs 8.2 months (HR 0.60)	Pivotal Phase III trial. Established pembrolizumab as the first-line standard of care. Superior, durable efficacy and significantly better safety vs chemo.	(89, 193)
IMbrave150	Unresectable HCC	Atezolizumab + Bevacizumab vs Sorafenib	15.6 months	OS: 19.2 vs 13.4 months (HR = 0.66), PFS: 6.9 vs 4.3 months (HR = 0.65)	Global landmark trial; established the first IO-based combo as a first-line standard; establishing a new paradigm for liver cancer treatment.	(100)
ORIENT-32	Unresectable HCC	Sintilimab + Bevacizumab biosimilar vs Sorafenib	10 months	OS: NR vs 10.4 months (HR = 0.57), PFS: 4.5 vs 2.8 months (HR = 0.56)	First Chinese-led Phase III immuno-combo study; demonstrated significant benefit in a population predominantly with HBV (94.5%) and prior TACE.	(194)
HIMALAYA	Unresectable HCC	Durvalumab + Tremelimumab (STRIDE) vs Sorafenib	16.1 months	OS: 16.5 vs 11.8 months (HR = 0.68); OS (Hong Kong and Taiwan): 29.4 vs 19.1 months (HR = 0.44)	Introduced a novel, single priming dose combo strategy; showed strong, durable benefit in Asian and HBV subpopulations; Durvalumab monotherapy was non-inferior to sorafenib.	(195, 196)

EC, esophageal cancer; GEJC, gastroesophageal junction cancer; GC, gastric cancer; MSI-H/dMMR mCRC, microsatellite instability-high or deficient mismatch repair colorectal cancer; HCC, hepatocellular carcinoma; OS, overall survival; PFS, progression-free survival; HR, hazard ratio; CPS, PD-L1 combined positive score; Chemo, chemotherapy; TP, paclitaxel + cisplatin; TACE, transcatheter arterial chemoembolization.

#### 3.1.1 The “hot” & responsive phenotype: high antigenicity and robust response

Tumors in this category are characterized by a high Tumor Mutational Burden (TMB) and abundant pre-existing T-cell infiltration. The primary barrier here is immune exhaustion; thus, releasing the checkpoint “brakes” yields significant efficacy.

The pinnacle of immunogenicity is exemplified by the subset of Colorectal Cancer (CRC) harboring deficient Mismatch Repair (dMMR) or high microsatellite instability (MSI-H). Due to defective repair machinery, these tumors generate a vast neoantigen load that naturally primes CD8^+^ T cells. Consequently, pembrolizumab monotherapy has established itself as the first-line standard, significantly surpassing chemotherapy in progression-free survival (KEYNOTE-177) (89). Strikingly, in the neoadjuvant setting, dual-ICI blockade (Nivolumab + Ipilimumab) has achieved pathological complete response (pCR) rates as high as 68% (NICHE-2), suggesting that for these highly “hot” tumors, chemotherapy may eventually become dispensable (90).

Similarly, Esophageal Squamous Cell Carcinoma (ESCC), prevalent in Asian populations, typically exhibits high somatic mutation rates and robust responsiveness. ICIs have successfully expanded from advanced lines to the adjuvant setting. The CheckMate-577 study demonstrated that adjuvant nivolumab doubles disease-free survival in patients with residual disease post-surgery (91). In the advanced first-line setting, combining PD-1 inhibitors (e.g., Nivolumab, Pembrolizumab, or domestic agents like Camrelizumab) with chemotherapy is now the standard of care, maximizing the anti-tumor potential of this immunogenic subtype (92–96).

#### 3.1.2 The “excluded” & modulatable phenotype: overcoming barriers via combination

In Gastric Cancer (GC) and Hepatocellular Carcinoma (HCC), effector T cells are often present at the invasive margin but are functionally inhibited or physically excluded by vascular and stromal barriers. Monotherapy is often insufficient; thus, combination strategies are required to breach these barriers and traffic T cells into the tumor core.

In Gastric Cancer, efficacy is closely tied to PD-L1 expression and HER2 status. Chemotherapy is an essential partner here, likely serving to induce Immunogenic Cell Death (ICD) and recruit T cells. This synergy was validated in landmark trials such as CheckMate-649 (Nivolumab + Chemo) (97) and KEYNOTE-859 (98), which confirmed significant survival benefits. For HER2-positive patients, the KEYNOTE-811 trial confirmed that adding pembrolizumab to the standard trastuzumab-chemotherapy regimen significantly enhances responses, establishing a new “Target-Immuno” precision standard (99).

Hepatocellular Carcinoma (HCC) presents a unique microenvironment defined by aberrant angiogenesis that suppresses immunity. Consequently, strategies combining ICIs with anti-angiogenic agents have replaced sorafenib as the first-line standard. The “T+A” regimen (Atezolizumab + Bevacizumab) demonstrated superior survival in the IMbrave150 trial (100). Mechanistically, anti-VEGF therapy normalizes tumor vasculature, facilitating T-cell infiltration and synergizing with PD-L1 blockade to overcome the immune-excluded state.

#### 3.1.3 The “cold” & refractory phenotype: the fortress of primary resistance

The vast majority of Colorectal Cancer (pMMR/MSS, ~95%) and Pancreatic Ductal Adenocarcinoma (PDAC) constitute the “immune desert” phenotype. These tumors are characterized by a paucity of neoantigens, dense desmoplasia, and a profound lack of effector T cells.

These malignancies serve as the quintessential models of primary resistance. Despite numerous trials, ICIs—whether as monotherapy or in combination with standard chemotherapy—have largely failed to improve survival compared to standard of care (101, 102). The dense stromal barrier (fibrosis) and the absence of priming signals render these tumors immunologically inert. Consequently, simply blocking PD-1 is futile. Current exploration focuses on “turning cold tumors hot” through TME remodeling. For instance, the UNION trial in MSS rectal cancer demonstrated that short-course radiotherapy could remodel the TME, sensitizing the tumor to ICI-chemotherapy combinations (103). This underscores that for “cold” phenotypes, the therapeutic priority is to ignite the immune cycle via remodeling strategies rather than mere checkpoint blockade.

### 3.2 Immunotherapy resistance in “cold” tumors: therapy-driven remodeling of immune specificity

While Section 2 elucidated chemoresistance mechanisms primarily supporting tumor-cell survival under cytotoxic stress, immunotherapy resistance in “cold” tumors—quintessentially pMMR/MSS colorectal cancer (CRC) and pancreatic ductal adenocarcinoma (PDAC)—is driven by active therapy-mediated immune editing. Under the selective pressure of immune checkpoint blockade (ICB), clones that remain antigenic and susceptible to effector-cell killing are eliminated, while subclones harboring reduced antigenicity or defective immune sensing are preferentially retained. Consequently, immune resistance is fundamentally shaped by the progressive erosion of recognition specificity, the uncoupling of recognition from cytotoxic execution, and the terminal exhaustion of tumor-reactive effectors.

#### 3.2.1 Antigenic narrowing and defective presentation under immune pressure

A hallmark of resistance is the dynamic narrowing of antigenic visibility rather than a mere static loss of machinery. The efficacy of ICIs necessitates the recognition of tumor neoantigens by CD8^+^ T cells via the MHC-I complex; however, resistant clones adopt a “stealth” phenotype to reduce immunogenic epitope display (104). This primary resistance in MSS CRC is dominated by MHC-I downregulation, often through genetic loss of the β2M gene (105, 106) or epigenetic silencing of the transactivator NLRC5 (107, 108). Recent single-cell analyses further reveal that transcription factors like SOX17 can directly repress MHC-I transcription, enforcing a state of immune invisibility distinct from classical mutational selection (109). These alterations specifically remodel the antigen-presenting landscape, allowing low-immunogenicity subclones to persist despite therapeutic intervention.

#### 3.2.2 Decoupling of immune recognition from cytotoxic execution

Even when recognition is preserved, the anti-tumor cycle may collapse if tumor cells become desensitized to effector cytokines. A critical layer of resistance lies in the uncoupling of antigen recognition from immune-mediated growth arrest. IFN-$\gamma$ signaling is essential for sustaining T-cell-mediated killing, yet resistant digestive tumors often develop profound insensitivity through loss-of-function mutations in JAK1/2 or IFNGR1. In CRC, the loss of OPTN (Optineurin) destabilizes the IFNGR1 receptor, dampening downstream STAT1 signaling and breaking the cytotoxic feedback loop (110). Furthermore, METTL3-mediated m6A modification is vital for stabilizing IFNGR1 transcripts; its dysregulation facilitates intrinsic resistance, though this may be reversible by METTL3 inhibitors (111, 112). Thus, resistance is characterized by a failure to translate T-cell encounters into effective cytotoxicity.

#### 3.2.3 Evolution of the terminal exhaustion phenotype

Resistance is also shaped by the epigenetic locking of T cells into dysfunctional states. The failure of PD-1 blockade in CRC and PDAC often reflects the co-expression of alternative inhibitory receptors—such as TIM-3, LAG-3, and TIGIT—creating a “whack-a-mole” scenario where single-agent blockade remains insufficient (113). TIGIT, for instance, directly impairs CD3^+^T-cell glucose metabolism, further compromising effector fitness (114). This exhaustion is enforced epigenetically; histone modifiers like HDAC3 promote the checkpoint B7x (B7-H4) through histone deacetylation, effectively paralyzing tumor-specific effectors (115). Resistance, therefore, represents a failure to maintain a functional, metabolic-fit pool of tumor-specific T cells under sustained therapeutic pressure.

#### 3.2.4 Microbiome-mediated shaping of systemic immune priming

Finally, the gut microbiome acts as an extrinsic regulator that governs the threshold of immune specificity. Dysbiosis in refractory CRC, often marked by Fusobacterium nucleatum enrichment, impairs efficacy by directly inhibiting NK and T-cell cytotoxicity via the Fap2-TIGIT interaction (116). Its metabolite, succinate, further restricts T-cell infiltration by suppressing the cGAS-STING-IFN-β pathway (117). Conversely, commensals like Bifidobacterium produce short-chain fatty acids (SCFAs) like butyrate, which act as HDAC inhibitors to enhance CD8^+^T-cell epigenetic memory and ICI durability (118). Thus, the microbiome reshapes the systemic and local environment to determine whether an anti-tumor response can be initiated or sustained.

Overall, immunotherapy resistance in cold tumors is not simply the result of isolated suppressive pathways. Rather, it reflects a dynamic process of immune selection under therapeutic pressure. Tumors gradually lose antigenic visibility, become less responsive to immune-mediated killing, and promote the dysfunction of tumor-reactive lymphocytes. Together, these alterations drive immune escape within an increasingly non-permissive microenvironment.

## 4 Interplay between chemotherapy and immunotherapy: from antagonism to synergy

**Figure 3:**
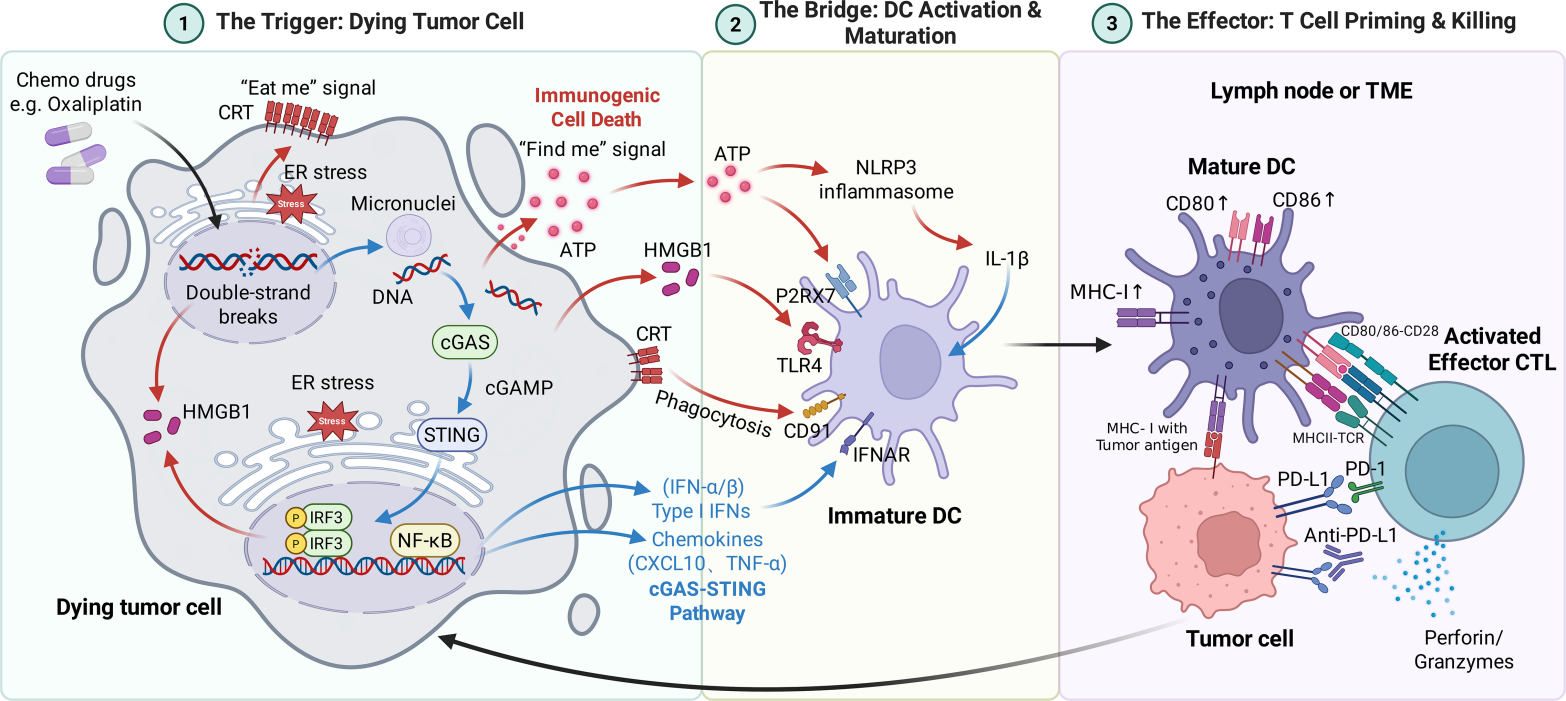
Schematic illustration of chemotherapy-induced ICD and cGAS-STING signaling in reshaping anti-tumor immunity. The process consists of three sequential phases: (1) The Trigger: Oxaliplatin induces DNA damage and ER stress, triggering the release of ICD mediators (surface CRT, extracellular ATP, nuclear HMGB1) and activating the cGAS-STING pathway to produce Type I interferons (IFN-α/β). (2) The Bridge: These danger signals recruit and mature Dendritic Cells (DCs) via specific receptor-ligand interactions: ATP-P2RX7, HMGB1-TLR4, CRT-CD91, and IFN-IFNAR. This leads to enhanced antigen processing and the upregulation of co-stimulatory markers (CD80/86). (3) The Effector: Licensed DCs cross-prime naïve CD8^+^ T cells. The resulting effector CTLs migrate to the tumor bed to mediate cytotoxicity. The integration of anti-PD-L1 blockade disrupts the PD-1/PD-L1 inhibitory axis, unleashing CTL activity and sustaining the cancer-immunity cycle. Figure created with BioRender.com.

As elucidated in the preceding sections, the mechanisms underpinning chemoresistance and innate immune resistance share a profound biological convergence within the tumor microenvironment (TME). Consequently, chemotherapy can no longer be viewed merely through the traditional lens of direct cytotoxicity and myelosuppression. Instead, emerging evidence reveals that chemotherapy exerts a “tunable” immunomodulatory effect that operates on a continuous spectrum ranging from antagonism to synergy. Deciphering this bidirectional regulation is the theoretical cornerstone for rational combinatorial design. Specifically, the interaction pivots on four critical axes: the induction of immunogenicity (the “Spark”), the triggering of adaptive resistance (the “Paradox”), the dynamic remodeling of the TME (the “Balance”), and the clinical management of overlapping toxicities (the “Constraint”). While the first three axes define the biological potential for synergy (A schematic summary of this process is provided in **Figure 3**), the fourth axis determines the pragmatic feasibility of these combinations in the clinical setting.

### 4.1 The “spark”: immunogenic cell death and cGAS-STING activation

The capacity of chemotherapy to bridge the gap between cytotoxic killing and adaptive immune activation is embodied in its ability to induce Immunogenic Cell Death (ICD). This process converts the tumor into an *in situ* vaccine via two parallel signaling pathways. First, specific chemotherapeutics—notably oxaliplatin (a bona fide ICD inducer superior to cisplatin in digestive cancers) and anthracyclines—trigger the spatiotemporal release of Damage-Associated Molecular Patterns (DAMPs) (119). The surface exposure of calreticulin (CRT) acts as an “eat me” signal for dendritic cells (DCs); extracellular ATP serves as a “find me” chemoattractant; and released HMGB1 binds to TLR4 to facilitate DC maturation (41, 120, 121). This cascade effectively reverses the antigen presentation defects discussed in Section 3, ensuring that tumor antigens are efficiently cross-presented to CD8^+^ T cells. Second, and perhaps more crucially for “cold” tumors, chemotherapy-induced DNA damage leads to the leakage of nuclear or mitochondrial DNA into the cytosol. This cytosolic DNA is sensed by cGAS, activating the STING pathway to induce Type I Interferon (IFN-I) production (122). This “viral mimicry” state inflames the TME, overcoming the immune exclusion barrier and recruiting T cells to the tumor core.

### 4.2 The “paradox”: adaptive immune resistance and PD-L1 upregulation

While chemotherapy-induced inflammation primes immunity, it simultaneously triggers a counter-regulatory feedback loop known as adaptive immune resistance. The secretion of IFN-γ by recruited T cells activates the JAK-STAT pathway in tumor cells, leading to the compensatory upregulation of surface PD-L1 expression (123). Historically viewed as a resistance mechanism, this phenomenon constitutes the strongest biological rationale for combining chemotherapy with ICIs. By upregulating PD-L1, chemotherapy effectively creates the target for PD-1/PD-L1 inhibitors, transforming a “low-target” tumor into a “high-target” one. This explains the clinical synergy observed in gastric cancer trials (e.g., CheckMate-649), where chemotherapy transiently opens a “therapeutic window” for ICIs to unleash their efficacy (97).

### 4.3 The “balance”: TME remodeling and lymphocyte dynamics

The net immunomodulatory outcome of chemotherapy hinges on the delicate balance between eliminating suppressive populations and preserving effector lymphocytes. This balance is strictly dose-dependent. High-dose “Maximum Tolerated Dose” (MTD) strategies often precipitate indiscriminate lymphodepletion and trigger a wound-healing response, where the release of CCL2 and CSF-1 recruits macrophages that rapidly polarize to the immunosuppressive M2 phenotype (70). In contrast, metronomic or optimized low-dose regimens can exert selective immunomodulation. Regulatory T cells (Tregs) and MDSCs are distinctively sensitive to agents like cyclophosphamide and gemcitabine due to their high proliferation rates (124). Consequently, optimized dosing can selectively deplete these suppressive barriers without compromising the effector T-cell pool (125, 126). Thus, the goal of modern combinatorial strategies is to define the “Optimal Biological Dose” (OBD)—a regimen designed to maximize ICD and antigen presentation while minimizing rebound immunosuppression.

### 4.4 The “constraint”: clinical safety and management of combined toxicity

The clinical translation from theoretical synergy to pragmatic intervention is fundamentally governed by the “Constraint” of overlapping toxicities. While the preceding axes—the Spark, the Paradox, and the Balance—define the biological potential for enhanced efficacy, the fourth axis determines the safety threshold that must not be breached. In the era of combinatorial therapy, the primary challenge lies in the synergistic toxicity that arises when chemotherapy-induced systemic insults intersect with immune-related adverse events (irAEs) (127). A critical manifestation of this constraint is the intersection of myelosuppression and lymphocyte dynamics. As discussed in the “Balance”, chemotherapy’s off-target effects, particularly the depletion of neutrophils and lymphocytes, can paradoxically extinguish the “Spark”. When chemotherapy-induced lymphopenia occurs concurrently with ICI-mediated organ inflammation, it creates a diagnostic and therapeutic dilemma: the high-dose corticosteroids required to manage severe irAEs may inadvertently suppress the very effector T-cell pool and cGAS-STING-mediated signals (the “Spark”) that the combination aimed to ignite.

In digestive system oncology, this constraint is further intensified by organ-specific cross-insult within the gastrointestinal (GI) tract. Mainstay agents such as 5-fluorouracil and oxaliplatin frequently cause significant mucosal injury, disrupting the intestinal epithelial barrier and the commensal microbiome interface (128, 129). This chemotherapy-induced “leaky gut” acts as a “second hit” that can lower the biological threshold for ICI-induced immune-mediated colitis, potentially escalating a manageable inflammatory response into a life-threatening complication. Such mucosal cross-insult often necessitates treatment interruptions, thereby closing the “therapeutic window” (the “Paradox”) created by initial immunogenic priming (130).

Ultimately, the goal of modern combinatorial design is to navigate these constraints by transitioning from the traditional “Maximum Tolerated Dose” (MTD) toward an OBD. By refining the sequencing and timing of administration—for instance, allowing sufficient recovery from myelosuppression before unleashing checkpoint blockade—clinicians can ensure that the “Spark” of anti-tumor immunity is sustained without succumbing to the “Constraint” of cumulative systemic failure.

## 5 Emerging combination strategies to overcome chemotherapy resistance

**Figure 4:**
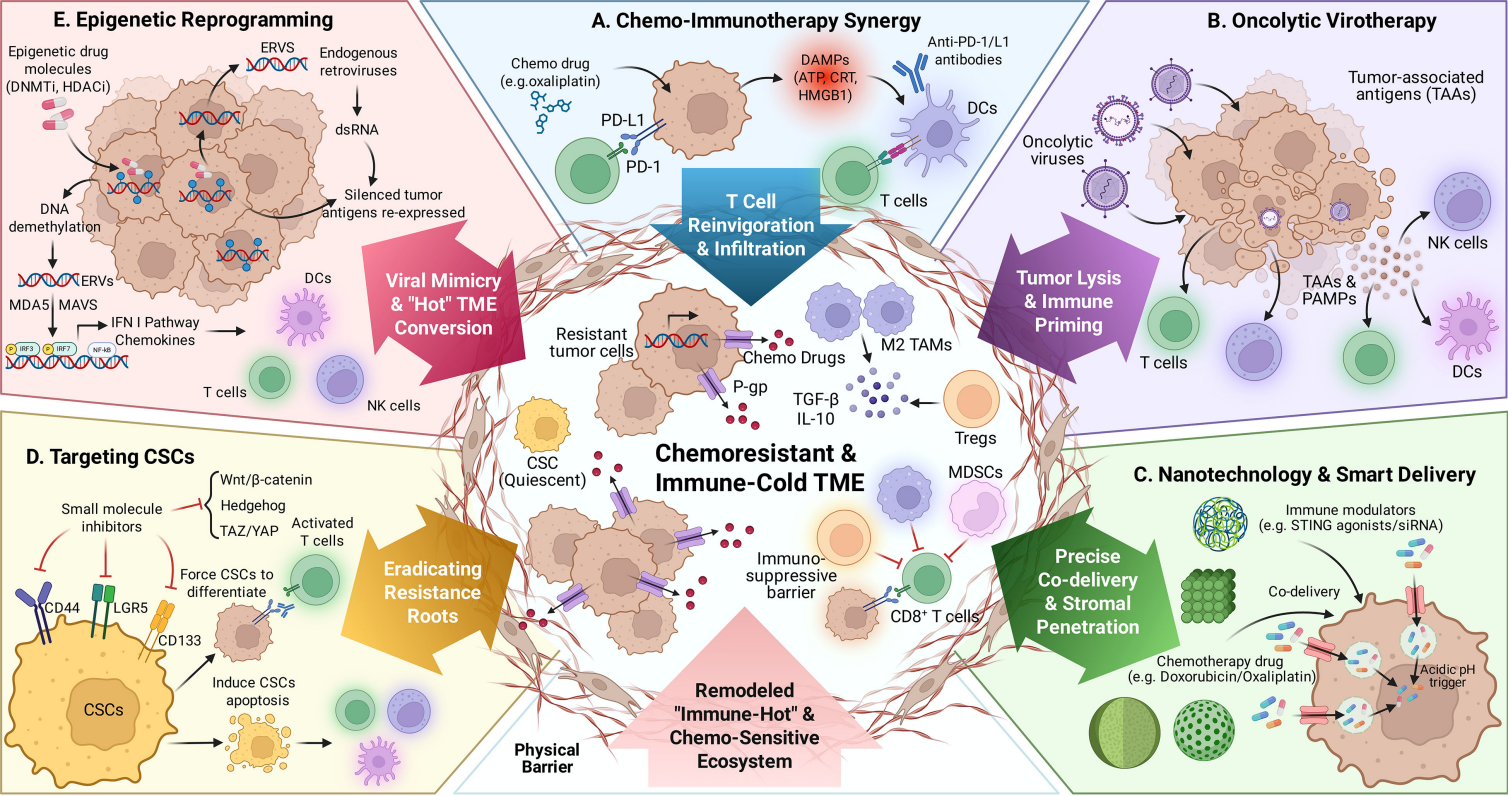
Strategic framework of emerging combinatorial interventions to dismantle the chemoresistant ecosystem. The Central Challenge: The center depicts the formidable landscape of a “Cold & Resistant” tumor. Resistance is driven by the convergence of intrinsic plasticity (drug efflux pumps, DNA repair, quiescent CSCs) and extrinsic barriers (desmoplastic stroma, immunosuppressive M2-TAMs/Tregs/MDSCs). Surrounding this hub are five complementary strategies designed to orchestrate a multimodal attack: **(A)** The Foundation (Chemo-Immunotherapy): Chemotherapeutic agents (e.g., oxaliplatin) function as immunological adjuvants to induce Immunogenic Cell Death (ICD). The release of DAMPs (ATP, CRT, HMGB1) primes innate sensing, while anti-PD-1/L1 antibodies exploit the resulting adaptive resistance to reinvigorate exhausted T cells. **(B)** The Breaker (Oncolytic Virotherapy): Oncolytic viruses act as “heavy artillery” to break tolerance. They selectively lyse tumor cells to release Tumor-Associated Antigens (TAAs) and PAMPs, dismantling both physical stromal barriers and immunological exclusion. **(C)** The Enabler (Nanotechnology): Multifunctional nanocarriers enable precise spatiotemporal co-delivery. By responding to TME-specific triggers (e.g., acidic pH/enzymes), they bypass efflux pumps and penetrate the dense stroma to deliver chemotherapeutics and immune modulators (e.g., STING agonists) to the tumor core. **(D)** The Root Eraser (CSC Targeting): Precision inhibitors disrupt key stemness signaling axes (e.g., Wnt/β-catenin, Hedgehog). This forces quiescent CSCs to differentiate or undergo apoptosis, effectively eradicating the cellular “reservoir” of recurrence. **(E)** The Primer (Epigenetic Reprogramming): Epigenetic drugs (DNMTi, HDACi) induce global demethylation to de-repress Endogenous Retroviruses (ERVs). This triggers a state of “Viral Mimicry” via dsRNA sensing, stimulating Type I IFN production to convert “cold” tumors into “hot” phenotypes. Figure created with BioRender.com.

Chemotherapy resistance remains the principal bottleneck limiting long-term survival in digestive system malignancies. As elucidated in the preceding sections, this phenomenon is not driven by a single pathway but by a sophisticated interplay between intrinsic cellular plasticity (e.g., DNA repair, stemness) and the extrinsic sanctuary of the tumor microenvironment (TME). Consequently, the traditional “one-target, one-drug” paradigm is increasingly inadequate to address this multifactorial challenge. To dismantle these entrenched barriers, a paradigm shift toward rational, mechanism-based combination strategies is essential. This section systematically explores the frontier of these synergistic strategies, which aim to simultaneously disrupt intrinsic survival signaling and reprogram the hostile TME. We begin with the optimization of standard chemo-immunotherapy, before advancing to next-generation interventions designed to rescue “cold” tumors, including epigenetic remodeling, oncolytic virotherapy, nanotechnology, and CSC-targeting. The strategic framework unifying these approaches is summarized in **Figure 4**, which provides a roadmap for precision intervention by targeting the chemoresistant ecosystem.

### 5.1 Chemo-immunotherapy: the foundation of synergistic intervention

While intrinsic and extrinsic resistance mechanisms pose formidable barriers, the strategic integration of chemotherapy and immune checkpoint inhibitors (ICIs) has established a new standard of care in digestive oncology. This synergy is not merely additive but multiplicative, predicated on chemotherapy functioning as an immunological adjuvant to “prime” the TME, “remodel” suppressive barriers, and “expose” vulnerable targets for immune attack.

Chemotherapy initiates the “upstream” events of the immunity cycle. As detailed in Section 4, specific agents (e.g., oxaliplatin, anthracyclines) act as bona fide inducers of Immunogenic Cell Death (ICD). By triggering the spatiotemporal release of DAMPs (CRT, ATP, HMGB1), chemotherapy effectively converts the tumor into an *in situ* vaccine. Crucially, chemotherapy-induced DNA damage activates the cGAS-STING pathway, providing the necessary Type I interferon signal to recruit and mature dendritic cells (DCs) (131, 132). This process transforms the “immune-cold” microenvironment into an “immune-hot” phenotype, creating the essential immunological substrate for ICIs.

Beyond activating innate immunity, chemotherapy exerts a “de-bulking” effect on the immunosuppressive stroma to lower the activation threshold for T cells. Optimized dosing regimens, such as low-dose cyclophosphamide or gemcitabine, can selectively deplete highly proliferative Regulatory T cells (Tregs) and MDSCs without compromising the effector pool (133). Concurrently, agents like taxanes have been shown to repolarize Tumor-Associated Macrophages (TAMs) from a pro-tumorigenic M2 phenotype toward a pro-inflammatory M1 phenotype in a TLR4-dependent manner (134). Furthermore, in hyper-vascular tumors like Hepatocellular Carcinoma (HCC), the combination of chemotherapy or anti-angiogenic agents (e.g., bevacizumab) normalizes the chaotic tumor vasculature. This structural remodeling reduces interstitial fluid pressure and facilitates the physical infiltration of CD8^+^ T cells into the tumor core, a mechanism central to the success of current first-line combinations (135–137).

Perhaps the most critical mechanism of synergy is the exploitation of Adaptive Immune Resistance. Chemotherapy-induced IFN-γ secretion triggers the compensatory upregulation of PD-L1 on tumor cells via the JAK-STAT pathway. While intrinsically a resistance mechanism, clinically this creates a transient “Therapeutic Window”. By administering anti-PD-1/L1 antibodies during this specific timeframe, clinicians can blockade this escape route, effectively intercepting the tumor’s attempt at resistance and converting a defensive maneuver into a therapeutic vulnerability (132).

Grounded in these mechanisms, chemo-immunotherapy has translated into significant survival benefits in Esophageal, Gastric, and Hepatocellular carcinomas. However, a critical bottleneck remains: in “immune-desert” tumors like Pancreatic Ductal Adenocarcinoma (PDAC) and pMMR/MSS Colorectal Cancer, traditional chemo-immunotherapy is often insufficient due to the profound lack of pre-existing immunity and dense physical barriers. This clinical ceiling necessitates the deployment of next-generation strategies—including epigenetic remodeling, oncolytic virotherapy, and nanotechnology—to artificially engineer immunogenicity where none exists.

### 5.2 Epigenetic remodeling: igniting viral mimicry to sensitize “cold” tumors

For tumors that remain refractory to standard chemo-immunotherapy—quintessentially the “immune-desert” phenotypes of pMMR/MSS CRC and PDAC—epigenetic dysregulation often serves as the master switch of immune evasion. Unlike genetic mutations which are irreversible, epigenetic silencing (e.g., DNA hypermethylation, histone deacetylation) is pharmacologically reversible. Consequently, epigenetic drugs are no longer viewed merely as cytotoxic agents but as “immunological primers” capable of reprogramming the transcriptional landscape to ignite an anti-tumor immune cycle in an otherwise inert microenvironment.

The most potent mechanism by which epigenetic agents reverse this inert state is “Viral Mimicry”. In resistant tumors, ancient Endogenous Retroviruses (ERVs) embedded in the genome are typically silenced by DNA hypermethylation to prevent immune detection. DNA Methyltransferase Inhibitors (DNMTis), such as 5-azacytidine and decitabine, induce global hypomethylation that “de-represses” these ERVs. The resulting accumulation of double-stranded RNA (dsRNA) is sensed by intracellular pattern recognition receptors (e.g., MDA5, RIG-I), mimicking a viral infection. This sensing event triggers a robust Type I Interferon (IFN-I) response via the cGAS-STING or MAVS pathways, creating a “pseudo-infected” inflammatory state that recruits cytotoxic T cells into the tumor core (24). In esophageal and colorectal cancers, this viral mimicry effect has been shown to overcome resistance to both chemotherapy and PD-1 blockade, effectively turning “cold” tumors “hot” (138–140).

Complementing this innate activation, epigenetic remodeling directly restores the adaptive recognition machinery. As highlighted in Section 3, the “Missing Self” phenotype (MHC-I downregulation) is a primary cause of resistance. Histone Deacetylase Inhibitors (HDACis), such as vorinostat and belinostat, promote an open chromatin structure at the promoters of MHC-I and Antigen Processing Machinery (APM) genes (e.g., TAP1, B2M). This restoration of antigen presentation renders the tumor visible again to cytolytic T cells (141, 142). Furthermore, emerging Histone Methyltransferase (HMT) inhibitors, targeting EZH2 or G9a, have been shown to prevent T-cell exhaustion and reshape the suppressive metabolic milieu, further reinforcing the durability of the immune response (143, 144).

Translating this biology into clinical practice, combinatorial strategies are yielding promising results. Trials in refractory CRC demonstrate that priming with DNMTis can sensitize patients to subsequent anti-PD-L1 therapy, achieving objective responses in historically non-responsive MSS populations (139, 140). Similarly, in gastric cancer, epigenetic priming not only suppresses proliferation but also restores sensitivity to 5-FU by reversing the methylation of key tumor suppressors (145, 146). Thus, epigenetic reprogramming acts as a critical “key” to unlock the immune gate, allowing subsequent therapies to function. However, the challenge remains to identify predictive biomarkers (e.g., basal ERV levels) to select patients who will benefit most from this priming strategy.

### 5.3 Oncolytic virotherapy: the “breaker” of physical and immunological barriers

If epigenetic remodeling acts as a molecular “primer, “ oncolytic viruses (OVs) function as the “heavy artillery” that dismantles refractory barriers within the tumor microenvironment (TME). These engineered agents have a dual mechanism: direct oncolysis via selective replication in cancer cells, followed by induction of systemic antitumor immunity. Critically, OVs exploit vulnerabilities associated with chemoresistance—such as impaired interferon signaling or defective antiviral sensing—to preferentially target and propagate within tumors (147, 148). This multifunctionality uniquely positions OVs to disrupt both the physical and immunological barriers that limit conventional therapies.

A key advantage of OVs is their ability to degrade the physical barrier of desmoplastic stroma. As noted earlier, the dense extracellular matrix (ECM) in pancreatic ductal adenocarcinoma (PDAC) acts as a “fortress, “ hindering drug perfusion and T-cell infiltration. OVs address this by carrying transgenes for matrix-remodeling enzymes. A prime example is the oncolytic adenovirus VCN-01, which expresses hyaluronidase to break down hyaluronan-rich ECM, reduce interstitial pressure, and improve drug perfusion (149). Clinical studies of VCN-01 combined with gemcitabine/nab-paclitaxel show intratumoral replication and encouraging activity, supporting the role of OVs in enhancing chemotherapy penetration in PDAC (150). This approach is vital for pancreatic cancer, where high stromal density, poor blood flow, and elevated pressure limit not only drug access but also viral entry and spread.

Beyond breaking stromal barriers, OVs convert immunologically “cold” tumors into inflamed sites. Viral lysis releases pathogen- and damage-associated molecular patterns (PAMPs/DAMPs) along with tumor antigens, creating a potent *in situ* vaccine (151). These signals recruit and activate dendritic cells more effectively than chemotherapy alone. OV infection can also repolarize macrophages toward an M1 phenotype, boost interferon and TNF-α signaling, and promote NK- and T-cell infiltration into immune-excluded regions (152). Thus, OVs do not simply kill tumor cells; they reprogram the local immune landscape to support antitumor immunity.

The translational promise of OVs lies in combination strategies. By inducing inflammation and sometimes upregulating PD-L1, OVs can sensitize tumors to immune checkpoint blockade. In KRAS-mutated colorectal cancer (CRC), pelareorep-based combinations show preliminary efficacy, suggesting viral-induced inflammation may overcome resistant immune phenotypes (153). OVs also synergize with radiotherapy by amplifying antigen release and immune priming (154). However, in digestive cancers, the efficacy of oncolytic virotherapy depends not only on immune stimulation but also on overcoming the distinct anatomical and physiological barriers of each target organ. In liver tumors, circulating viruses are readily sequestered by the hepatic reticuloendothelial system, including Kupffer cells and sinusoidal endothelial components, which can reduce productive tumor delivery and increase non-productive hepatic uptake (155, 156). In PDAC, dense desmoplastic stroma, poor perfusion, and elevated interstitial pressure limit not only drug penetration but also viral entry and intratumoral spread (157). In CRC, the mucosal interface and local microbiota may influence viral stability, persistence, and spread within the tumor niche, while local innate antiviral signaling may further restrict productive infection and treatment response (158–160).

A balanced view must also address safety. Common adverse events are flu-like (fever, chills, fatigue). However, severe systemic inflammatory reactions—including cytokine release syndrome (CRS)-like or sepsis-like events—have been reported, especially in combinations (161–163). Because CRS can mimic infection, post-treatment fever and organ dysfunction require careful evaluation. Additionally, tumor selectivity is not absolute. While genetic modifications (e.g., E1B-55K deletion in adenovirus) enhance cancer specificity, some off-target sequestration in liver and spleen remains possible, raising toxicity concerns (164).

OVs also pose distinct clinical pharmacology challenges. As replicating agents, their biologically effective dose is dynamic, not fixed. It depends on administration route, tumor permissiveness, replication kinetics, pre-existing antibodies, immune clearance, and stromal resistance (165). Thus, dose escalation alone cannot define optimal use. Rational development requires parallel assessment of viral kinetics, biodistribution, intratumoral spread, and shedding—particularly for systemic delivery in liver-involved cancers. In summary, while OVs are a promising adjunct to chemo-immunotherapy in digestive cancers, their broader integration will require advances in vector design, organ-specific delivery strategies, and proactive safety monitoring.

### 5.4 Targeting plasticity: eradicating the cellular roots of resistance

While epigenetic priming and oncolytic “breaching” address the tumor microenvironment, long-term remission requires eliminating the “Cellular Root” of resistance—the Cancer Stem Cells (CSCs). Situated at the apex of the intratumoral hierarchy, CSCs act as a reservoir for recurrence and metastasis. Characterized by metabolic quiescence, high expression of drug efflux pumps (ABC transporters), and robust DNA repair capacity, CSCs exhibit intrinsic recalcitrance to conventional chemotherapy. Consequently, even after effective debulking of the tumor mass, surviving CSCs can repopulate the tumor, often emerging with a more aggressive phenotype. Therefore, a curative strategy must specifically target the molecular machinery of stemness and dismantle the CSC-specific immune sanctuary.

The primary strategy involves dismantling the signaling pathways that maintain CSC self-renewal. CSCs are critically dependent on developmental pathways—such as Wnt/β-catenin, Hedgehog, and YAP1/TAZ—to sustain their undifferentiated state (8). Targeting these vulnerabilities offers a precise therapeutic avenue. For instance, PRI-724, a specific inhibitor of the CBP/β-catenin interaction, effectively disrupts the sphere-forming ability of gastric and colorectal CSCs, sensitizing them to chemotherapy (166, 167). Similarly, targeting the Hippo pathway with Verteporfin (a YAP1 inhibitor) significantly impairs tumorigenicity and metastatic potential (168). By blocking these “stemness signals”, these agents force CSCs to differentiate into a more chemosensitive lineage, stripping them of their protective plasticity.

Crucially, CSCs are not merely chemo-resistant; they are masters of immune evasion. They reside in “immune-privileged” niches, actively downregulating MHC-I molecules to evade T-cell recognition while upregulating checkpoints like PD-L1 and CD47 (“don’t eat me” signal) to paralyze immune effectors. Therefore, combining CSC-targeting with immunotherapy offers a synergistic opportunity. Immune checkpoint inhibitors can enhance the elimination of CSCs that might otherwise survive chemotherapy (29). Furthermore, novel metabolic-immune combinations, such as Vitamin D analogs combined with anti-PD-1, have been shown to remodel the stromal niche, effectively exposing CSCs to immune surveillance (169).

To achieve precision in eradication, advanced delivery systems are essential. Nanotechnology plays a pivotal role here by utilizing CSC-specific surface markers (e.g., CD44, CD133, LGR5) for targeted delivery. For example, CD44v6-targeted polymeric micelles co-delivering niclosamide and oxaliplatin have demonstrated the ability to specifically ablate the CSC population in colorectal cancer, inhibiting metastasis (170). Looking forward, the integration of CRISPR/Cas9 gene editing to knockout survival genes (e.g., Survivin) and the use of Patient-Derived Organoids (PDOs) for drug screening represent the frontier of precision oncology (171). By systematically eradicating these roots, we aim to transform chemotherapy from a temporary “pruning” measure into a potentially curative intervention.

### 5.5 Nanotechnology: an organ-adapted enabler of precision co-delivery and TME remodeling

While the preceding strategies—ranging from chemo-immunotherapy to epigenetic priming and CSC targeting—offer potent mechanisms to overcome resistance, their translational success is frequently thwarted by pharmacokinetic discordance, off-target toxicities, and heterogeneous intratumoral access (172, 173). In this context, nanotechnology should not be regarded merely as a passive drug-loading platform, but as a delivery engineering strategy tailored to the unique physiological barriers of each digestive organ. The value of nanotechnology in digestive oncology lies in its ability to align therapeutic payloads with organ-specific transport biology: navigating hepatic sequestration in liver tumors, breaching the desmoplastic fortress in pancreatic cancer, and negotiating the complex biotic-abiotic interface in the colorectum.

In liver-directed therapy, the inherent tendency of nanoparticles to accumulate in the liver is a “double-edged sword”. Following systemic administration, the majority of nanocarriers are rapidly sequestered by the Mononuclear Phagocyte System (MPS), specifically Kupffer cells and liver sinusoidal endothelial cells (LSECs) (174). For hepatocellular carcinoma (HCC), the engineering priority is to distinguish productive tumor targeting from premature phagocytic clearance. This necessitates the deployment of “liver-aware” nanoplatforms that utilize biomimetic coatings or size-optimized designs to evade filtration and exploit the unique hepatic sinusoidal fenestrae for enhanced tumor-selective uptake (175).

In pancreatic ductal adenocarcinoma (PDAC), the primary bottleneck shifts to the desmoplastic stroma. The dense deposition of collagen and hyaluronan, coupled with high interstitial fluid pressure (IFP), creates a physical sanctuary that excludes most therapeutic agents (176). Here, nanotechnology must function as a penetration-first tool. Effective delivery requires matrix-responsive or size-transformable designs that can dynamically adapt to the physical exclusion of the PDAC microenvironment, ensuring that co-delivery of chemotherapeutics and immunomodulators reaches the previously inaccessible tumor core (177).

For colorectal cancer (CRC), nanomedicine must negotiate a multi-layered barrier comprising luminal pH gradients, thick mucus layers, and a diverse local microbiota. Rather than passive obstacles, these features serve as environmental triggers for site-selective release. This ecological context makes CRC an ideal candidate for “microbiota-aware” nanoplatforms that integrate tumor targeting with the metabolic cues of the gut environment, thereby synchronizing drug release with the optimal therapeutic window (178, 179).

Grounded in this organ-adapted framework, nanotechnology enables the sophisticated coordination of combination therapies. In HCC, calcium carbonate nanoparticles co-delivering doxorubicin and the PCSK9 inhibitor evolocumab (DECP) triggered robust immunogenic cell death (ICD) while concurrently depleting intratumoral Tregs, thereby sensitizing the tumor to anti-PD-1 therapy (180). In the refractory PDAC environment, a size-switchable nanosystem co-delivering vactosertib and paclitaxel effectively anchored to tumor-associated fibronectin and released smaller nanospheres to inhibit TGF-β1-driven matrix hyperplasia, significantly enhancing stromal penetration (181). Furthermore, nanodrug-bacteria conjugates have exploited hypoxia tropism to reach the PDAC parenchyma and degrade collagen, disrupting the integrin α3β1-FAK signaling axis to synergize with checkpoint blockade (182). In the colorectal niche, ATP/pH dual-responsive ZIF-90 nanoparticles achieved lysosomal-selective release and bypassed P-glycoprotein-mediated efflux, addressing cellular resistance once anatomical access was secured (183). Microbiota-oriented strategies, such as orally available dextran-aspirin or prebiotic capecitabine-loaded nanoparticles, have demonstrated that modulating the microbial ecosystem can directly enhance the durability of αPD-L1 therapy in CRC models (184, 185).

Despite these breakthroughs, the transition of nano-combinatorial strategies from bench to bedside remains constrained by manufacturing complexity, batch-to-batch reproducibility (CMC), and long-term biocompatibility. Future progress will depend on the convergence of AI-driven design and high-fidelity models like Patient-Derived Organoids (PDOs) to predict nanoparticle-organ interactions. Ultimately, nanotechnology is poised to be the final piece of the puzzle, translating mechanistic synergies into anatomically precise clinical cures.

## 6 Future perspectives

Despite the promising landscape of combinatorial strategies described herein, their translation from bench to bedside remains challenging. This difficulty mainly stems from the lack of precise predictive models and the complexity of tumor evolution. Bridging this gap requires a fundamental shift toward deep phenotyping and the integration of advanced technologies.

The first critical frontier is the “Spatial Revolution”. We must move beyond bulk sequencing and make greater use of single-cell multi-omics and spatial transcriptomics. By mapping the “resistance ecosystem” at cellular resolution, we can identify rare drug-tolerant subclones and visualize the spatial exclusion of T cells. Such high-resolution profiling can reveal patient-specific vulnerabilities, including stromal barriers or metabolic niches, that are often invisible to traditional pathology. This, in turn, may support more precise target selection.

Complementing this biological depth is the need for advanced modeling and digitization. The current trial-and-error approach in the clinic is inefficient. Future efforts should focus on integrating patient-derived organoids (PDOs) with artificial intelligence (AI) to create “clinical avatars” or “digital twins”. These high-fidelity models may recapitulate the complex TME ex vivo and allow high-throughput screening of combinatorial dosing schedules and sequencing strategies before treatment begins. In parallel, AI algorithms can help interpret complex multi-omics data, predict overlapping toxicities, and identify non-intuitive synergistic partners. Together, these advances may accelerate the drug development pipeline.

Finally, resistance management must evolve from a static snapshot to dynamic tracking. Chemoresistance is an evolutionary process driven by selective pressure, and a single pre-treatment biopsy is therefore insufficient. Future clinical protocols should incorporate longitudinal monitoring through liquid biopsies, such as ctDNA and circulating exosomes, together with microbiome profiling. This “dynamic precision” approach may allow clinicians to detect resistant clones or immune escape variants in real time. It may also support adaptive changes in therapy, such as introducing an epigenetic primer or an oncolytic breaker at the point when the tumor begins to evolve. In this way, treatment may stay one step ahead of disease progression.

## 7 Conclusion

Chemoresistance in digestive system malignancies remains a major bottleneck that limits long-term survival. As discussed throughout this review, resistance is not simply a cellular refusal to die. Rather, it is a sophisticated ecological adaptation shaped by the interplay between intrinsic cellular plasticity and an extrinsic, fortified tumor microenvironment (TME). The failure of traditional monotherapies highlights a fundamental reality: linear interventions are unlikely to dismantle a multidimensional resistance network. Accordingly, the future of oncology lies not in finding a more potent cytotoxic agent, but in deciphering and disrupting the complex crosstalk between tumors and their immune ecosystem.

To overcome these entrenched barriers, we have outlined a shift toward mechanism-based combinatorial strategies. This approach goes beyond simple additivity and aims to achieve true biological synergy through a multimodal therapeutic framework. Optimized chemo-immunotherapy serves as the foundation by leveraging immunogenic cell death (ICD) to prime the immune cycle. In refractory “cold” tumors, epigenetic modulators may function as molecular primers that induce viral mimicry. At the same time, oncolytic viruses and CSC-targeting agents can serve as physical and cellular “breakers” to disrupt stromal barriers and eliminate the roots of recurrence. Supporting these modalities, nanotechnology acts as a critical enabler by ensuring precise and spatiotemporally coordinated delivery of therapeutic payloads.

In summary, the treatment paradigm for digestive system tumors is shifting from indiscriminate cytotoxicity to comprehensive ecosystem reprogramming. By integrating immunology, biotechnology, and data science, the combinatorial strategies highlighted in this review provide a promising roadmap for precision intervention. Ultimately, these advances may help transform chemoresistance from a fatal endpoint into a manageable challenge and improve the survival outlook for patients worldwide.
